# Mulheres de Meia-Idade — Um Grupo de Alto Risco para Mortalidade Pós-Infarto do Miocárdio

**DOI:** 10.36660/abc.20250701

**Published:** 2026-01-07

**Authors:** Maria Antonieta Albanez A. de Medeiros Lopes, Mayara Viana, Júlia Nóbrega, Heitor N. Albanez A. de Medeiros, Gláucia Maria Moraes de Oliveira

**Affiliations:** 1 Real Hospital Português Recife PE Brasil Real Hospital Português, Recife, PE – Brasil; 2 Hospital São Marcos Recife PE Brasil Hospital São Marcos, Recife, PE – Brasil; 3 Universidade Federal do Maranhão Hospital Universitário São Luís MA Brasil Hospital Universitário da Universidade Federal do Maranhão, São Luís, MA – Brasil; 4 Universidade Federal do Rio de Janeiro Rio de Janeiro RJ Brasil Universidade Federal do Rio de Janeiro, Rio de Janeiro, RJ – Brasil

**Keywords:** Mulheres, Mortalidade, Infarto

**Figura 1:**
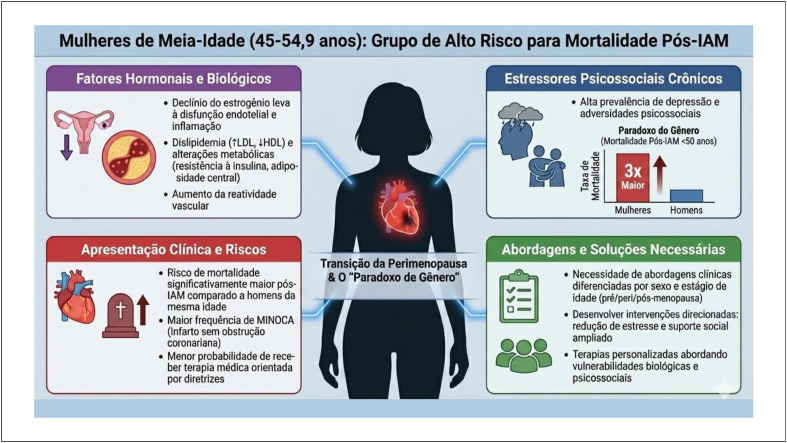
Fatores de risco e mecanismos associados ao aumento da mortalidade pós-infarto agudo do miocárdio em mulheres de meia-idade (45-54,9 anos). A figura ilustra a complexa interação entre fatores hormonais e biológicos (declínio do estrogênio, dislipidemia, alterações metabólicas e aumento da reatividade vascular), estressores psicossociais crônicos (alta prevalência de depressão e adversidades psicossociais) e suas consequências clínicas durante a transição da perimenopausa. O "Paradoxo de Gênero" é evidenciado pela mortalidade pós-IAM aproximadamente três vezes maior em mulheres comparadas a homens da mesma faixa etária.

As doenças cardiovasculares (DCV) continuam sendo a principal causa de morte no mundo, com o infarto agudo do miocárdio (IAM) respondendo por significativa morbidade e mortalidade. Apesar das importantes reduções na mortalidade cardiovascular em geral, persistem disparidades de gênero, que afetam particularmente as mulheres em diferentes contextos demográficos.^1^ No presente mini editorial, destacamos evidências recentes provenientes do estudo original realizado no Sistema Único de Saúde em Curitiba, Brasil, que acompanhou 4.896 pacientes hospitalizados por IAM entre 2008 e 2015.^2^ Esse estudo pioneiro demonstrou que mulheres de meia-idade (45 a 54,9 anos) apresentam risco significativamente maior de mortalidade pós-infarto em comparação a homens da mesma faixa etária, independentemente de fatores clínicos ajustados. Esses dados refletem um importante alerta para a necessidade de abordagens clínicas diferenciadas e específicas para esse grupo (Figura 1). A análise da coorte pública brasileira fortalece as evidências internacionais sobre o paradoxo de gênero nas DCV e reforça o papel crítico das alterações hormonais e dos determinantes psicossociais no agravamento do prognóstico feminino pós-IAM. Isso contradiz a suposição comum de que mulheres nessa faixa etária mantêm uma proteção natural cardiovascular, levantando questões críticas sobre a atenção dada à saúde cardiovascular nessa fase importante da vida.^2^

O chamado "paradoxo de gênero" nas DCV é bem estabelecido, especialmente entre mulheres mais jovens. Estudos mostram que mulheres com menos de 50 anos têm quase três vezes maior mortalidade após IAM do que homens da mesma faixa etária, independentemente do histórico médico prévio, gravidade da doença ou tipo de tratamento.^3,4^ Esse paradoxo é notável, dado que mulheres de meia-idade frequentemente apresentam menos fatores tradicionais de risco cardiovascular e tendem a ter melhores resultados após intervenção coronariana percutânea.^5^ Os achados do estudo^2^ estão alinhados com dados globais e nacionais que apontam que as mulheres desenvolvem IAM aproximadamente 5 anos depois dos homens (idade média 65,1 vs. 60,3 anos), mas o efeito protetor do sexo feminino diminui e se inverte na faixa etária de 45 a 54,9 anos.^5^ Esse período coincide com a perimenopausa e o início da menopausa, ressaltando a importância das alterações hormonais no aumento da suscetibilidade cardiovascular.^6^

Uma complexa interação de mecanismos biológicos explica o aumento do risco cardiovascular entre mulheres perimenopáusicas. A transição para a menopausa resulta em declínio gradual na produção ovariana de estrogênio. A redução dos estrogenos leva à dislipidemia, caracterizada por aumento do colesterol LDL (lipoproteína de baixa densidade) e redução do colesterol HDL (lipoproteína de alta densidade), promove disfunção endotelial, prejudicando a vasodilatação mediada por óxido nítrico e aumentando a reatividade vascular e a inflamação.^6,7^ Adicionalmente, ocorrem distúrbios metabólicos como hiperinsulinemia, resistência à insulina, adiposidade central e elevação da pressão arterial, impulsionando o desenvolvimento da doença arterial coronariana (DAC).^6-8^

As mudanças biológicas e hormonais influenciam a diversidade das apresentações isquêmicas em mulheres na meia-idade. Enquanto a DAC é mais prevalente entre mulheres pré-menopáusicas de alto risco com diabetes, obesidade e hipertensão, mulheres perimenopáusicas apresentam com mais frequência infarto do miocárdio com artérias coronárias não obstrutivas (MINOCA), representando aproximadamente 1–15% dos casos de IAM ou síndrome coronariana aguda — e as mulheres são cinco vezes mais propensas que os homens a desenvolver MINOCA.^9,10^

Mulheres perimenopáusicas frequentemente apresentam menos fatores de risco cardiovascular convencionais em comparação aos pacientes com DAC obstrutiva. Elas predominam em síndromes coronarianas agudas sem elevação do segmento ST e têm menor probabilidade de serem submetidas à revascularização ou receber terapia médica orientada pelas diretrizes após o IAM.^10,11^

O MINOCA não é benigno, e pacientes afetadas podem ter complicações graves, incluindo parada cardíaca, redução da fração de ejeção do ventrículo esquerdo e insuficiência cardíaca. Embora a mortalidade em pacientes com MINOCA seja menor que a relacionada ao IAM com DAC obstrutiva, ainda é substancialmente maior do que em coortes saudáveis pareadas. As diretrizes atuais enfatizam tratamento individualizado baseado na etiologia para reduzir morbidade e mortalidade.^11^

O aumento do risco cardiovascular em mulheres de meia-idade não pode ser explicado apenas por fatores biológicos. Depressão e adversidades psicossociais são especialmente prevalentes nesse grupo, incluindo dificuldades socioeconômicas, estresse de cuidadora e violência por parceiro íntimo, um estressor crônico significativo que afeta os desfechos de saúde das mulheres. Essa sensibilidade aumentada ao estresse é atribuída às diferenças sexuais na reatividade vasomotora e nas respostas inflamatórias A isquemia miocárdica induzida por estresse mental agudo ocorre com maior frequência em mulheres, especialmente de meia-idade, em comparação aos homens.^8^

As diretrizes clínicas devem ser refinadas para integrar perfis de risco específicos por sexo e idade, contemplando as fases pré-menopausa, perimenopausa e pós-menopausa. Pesquisas futuras devem desenvolver e avaliar intervenções direcionadas como programas de redução de estresse, suporte social ampliado e terapias personalizadas que abordem as vulnerabilidades biológicas e psicossociais singulares dessa população.
