# Supplementary material for: Mulheres de Meia-Idade — Um Grupo de Alto Risco para Mortalidade Pós-Infarto do Miocárdio
Source: Arq Bras Cardiol. 2026 Jan 7;122(10):e20250701. [Article in Portuguese] doi: 10.36660/abc.20250701 (PMC12978168; doi:10.36660/abc.20250701)
Supplement: Translation

# Translation: Middle-Aged Women — A High-Risk Group for Post-Myocardial Infarction Mortality

**DOI:** 10.36660/abc.20250701i

**Authors:** Maria Antonieta Albanez A. de Medeiros Lopes, Mayara Viana, Júlia Nóbrega, Heitor N. Albanez A. de Medeiros, Gláucia Maria Moraes de Oliveira

**Affiliations:** 1 Recife PE Brazil Real Hospital Português, Recife, PE – Brazil; 2 Recife PE Brazil Hospital São Marcos, Recife, PE – Brazil; 3 São Luís MA Brazil Hospital Universitário da Universidade Federal do Maranhão, São Luís, MA – Brazil; 4 Rio de Janeiro RJ Brazil Universidade Federal do Rio de Janeiro, Rio de Janeiro, RJ – Brazil

**Keywords:** Women, Mortality, Infarction

Cardiovascular diseases (CVD) remain the leading cause of death worldwide, with acute myocardial infarction (MI) accounting for significant morbidity and mortality. Despite substantial reductions in overall cardiovascular mortality, gender disparities persist, particularly affecting women in different demographic contexts.^1^ In this mini-editorial, we highlight recent evidence from an original study conducted in the Brazilian Unified Health System (SUS) in Curitiba, Brazil, which followed 4,896 patients hospitalized for acute MI between 2008 and 2015.^2^ This pioneering study demonstrated that middle-aged women (45 to 54.9 years) have a significantly higher risk of post-infarction mortality compared to men of the same age group, regardless of adjusted clinical factors. These data provide a critical warning about the need for differentiated, specific clinical approaches for this group (Figure 1). The analysis of the Brazilian public cohort strengthens international evidence on the gender paradox in CVD. It reinforces the critical role of hormonal changes and psychosocial determinants in worsening the prognosis for women after MI. This contradicts the common assumption that women in this age group maintain natural cardiovascular protection, raising critical questions about the attention given to cardiovascular health at this vital stage of life.^2^

**Figure 1:** Risk factors and mechanisms associated with increased post-acute myocardial infarction mortality in middle-aged women (45-54.9 years). The figure illustrates the complex interaction between hormonal and biological factors (estrogen decline, dyslipidemia, metabolic changes, and increased vascular reactivity), chronic psychosocial stressors (high prevalence of depression and psychosocial adversities), and their clinical consequences during the perimenopausal transition. The &quot;Gender Paradox&quot; is evidenced by approximately three times higher post-MI mortality in women compared to men of the same age group.

The so-called "gender paradox" in CVD is well established, especially among younger women. Studies show that women under 50 have almost three times higher mortality after MI than men of the same age group, regardless of previous medical history, disease severity, or type of treatment.^3,4^ This paradox is remarkable, given that middle-aged women often have fewer traditional cardiovascular risk factors and tend to have better outcomes after percutaneous coronary intervention.^5^ The study^2^ findings are aligned with global and national data that indicate that women develop MI approximately 5 years later than men (mean age 65.1 vs. 60.3 years). Still, the protective effect of the female sex diminishes and reverses in the 45-54.9 age range.^5^ This period coincides with perimenopause and the onset of menopause, highlighting the importance of hormonal changes in increasing cardiovascular susceptibility.^6^

A complex interaction of biological mechanisms explains the increased cardiovascular risk among perimenopausal women—the transition to menopause results in a gradual decline in ovarian estrogen production. Reduced estrogen levels lead to dyslipidemia, characterized by increased LDL (low-density lipoprotein) cholesterol and reduced HDL (high-density lipoprotein) cholesterol, which promotes endothelial dysfunction, impairs nitric oxide-mediated vasodilation, and increases vascular reactivity and inflammation.^6,7^ Additionally, metabolic disorders such as hyperinsulinemia, insulin resistance, central adiposity, and elevated blood pressure occur, driving the development of coronary artery disease (CAD). ^6-8^

Biological and hormonal changes influence the diversity of ischemic presentations in middle-aged women. While CAD is more prevalent among high-risk premenopausal women with diabetes, obesity, and hypertension, perimenopausal women more frequently present with myocardial infarction with non-obstructive coronary arteries (MINOCA), representing approximately 1–15% of cases of acute MI or acute coronary syndrome, and women are five times more likely than men to develop MINOCA.^9,10^

Perimenopausal women often have fewer conventional cardiovascular risk factors compared to patients with obstructive CAD. They predominate in acute coronary syndromes without ST-segment elevation and are less likely to undergo revascularization or receive guideline-directed medical therapy after MI.^10,11^

MINOCA is not benign, and affected patients may experience serious complications, including cardiac arrest, reduced left ventricular ejection fraction, and heart failure. Although mortality in patients with MINOCA is lower than that related to acute MI with obstructive CAD, it is still substantially higher than in matched healthy cohorts. Current guidelines emphasize individualized treatment based on etiology to reduce morbidity and mortality.^11^

The increased cardiovascular risk in middle-aged women cannot be explained solely by biological factors. Depression and psychosocial adversities are especially prevalent in this group, including socioeconomic difficulties, caregiver stress, and intimate partner violence, a significant chronic stressor that affects women's health outcomes. This sensitivity, increased age-related stress, is attributed to sex differences in vasomotor reactivity and inflammatory responses. Myocardial ischemia induced by acute mental stress occurs more frequently in women, especially middle-aged women, compared to men.^8^

Clinical guidelines should be refined to integrate sex- and age-specific risk profiles, encompassing the pre-menopausal, perimenopausal, and postmenopausal phases. Future research should develop and evaluate targeted interventions such as stress reduction programs, expanded social support, and personalized therapies that address the unique biological and psychosocial vulnerabilities of this population.
